# Improving the Concentrations of the Active Components in the Herbal Tea Ingredient, *Uraria crinita*: The Effect of Post-harvest Oven-drying Processing

**DOI:** 10.1038/srep38763

**Published:** 2017-01-12

**Authors:** Jung Chao, Yuntao Dai, Hao-Yuan Cheng, Wing Lam, Yung-Chi Cheng, Ke Li, Wen-Huang Peng, Li-Heng Pao, Ming-Tsuen Hsieh, Xue-Mei Qin, Meng-Shiou Lee

**Affiliations:** 1 Institute of Pharmacology, National Yang-Ming University, College of Medicine, Taipei, Taiwan; 2 Institute of Chinese Materia Medica, China Academy of Chinese Medical Sciences, Beijing, China; 3 Department of Nursing, Chung-Jen Junior College of Nursing, Health Sciences and Management, Chia-Yi, Taiwan; 4 Department of Pharmacology, Yale University School of Medicine, New Haven, Connecticut, United States; 5 Modern Research Center for Traditional Chinese Medicine of Shanxi University, Shanxi, China; 6 Department of Chinese Pharmaceutical Sciences and Chinese Medicine Resources, China Medical University, Taiwan; 7 Research Center for Industry of Human Ecology, Graduate Institute of Health-Industry Technology and Department of Nutrition and Health Sciences, Chang Gung University of Science and Technology, Kweishan, Taoyuan, Taiwan; 8 School of Pharmacy, National Defense Medical Center, Taipei, Taiwan

## Abstract

*Uraria crinita* is widely used as a popular folk drink; however, little is known about how the post-harvest operations affect the chemical composition and bioactivity of UC. We assessed three drying methods (Oven-drying, Air-drying, Sun-drying), as well as the Oven-drying temperature using metabolomics approaches and bioactivity assays. The samples processed at 40 degree show a greater effect on the levels of estrogen receptor-alpha activity and nuclear factor erythroid 2–related factor 2 activity, anti-oxidative activity, and cyclooxygenase-2 inhibition compared with the other samples. A multivariate analysis showed a clear separation between the 40 degree Oven-dried samples and the other samples, which is consistent with the results of bioactivity assay. These results are ascribed to at least two-fold increase in the concentrations of flavonoids, spatholosineside A and triterpenoids in the oven-dried samples compared with the other groups. The proposed Oven-drying method at 40 degree results in an improved quality of UC.

The rigorous implementation of Good Agricultural and Collection Practices (GACP) and Good Manufacturing Practices (GMP) is a key step towards improving the quality of herbal teas or functional foods and ensuring the safety, efficacy, and consistent quality of the final products^1^. However, the current quality control approaches for herbal tea are regional and partial. Different agricultural and manufacturing practices are employed in different places that process the same herbal tea, and this is likely to be one of the major reasons for the heterogeneous quality of the final products. Diverse effects on the nutrient components of different materials may be induced during food processing. Previous study have shown that the retention factor (RF) has been used to calculate the polyphenol contents in processed food: an RF < 1 indicates a reduced polyphenol content in the processed food, whereas RF = 1 and RF > 1 indicate full retention or an increase, respectively^2^. However, most studies on the effect of post-harvest processing are focused on some targeted components. Therefore, to achieve an overall improvement in quality, the impact of these practices must be considered. Appropriate research is needed to deepen our knowledge of the processing methods and standard operating procedures; this knowledge will enhance the regulation and quality of crude herbal teas.

*Uraria crinita* (UC) (Leguminosae) is a traditional edible plant in Taiwan and China. The aqueous extract of UC, commonly known as “ginseng-like” herbal tea, is a popular folk drink or functional food^3^. The root is traditionally used to regulate digestive activity, for deworming, and to treat diarrhea^3^,^4^,^5^. UC is also used by herbalists and doctors for its detoxifying action, its ability to remove swelling^5^ and its antitussive effects^3^. A UC ethanol extract is used in herbal cuisine and as part of a medicated diet^3^,^4^,^5^. Pharmacological investigations have demonstrated that plants of the *Uraria* genus have a wide range of biological activities, including anti-inflammatory, analgesic^6^, and antimicrobial activities^7^, as well as cytotoxicity^8^. Previous studies have shown that UC exhibits anti-oxidative activity, nitric oxide-scavenging activity^9^ and the ability to repel and kill blowfly larvae^10^.

Our fieldwork has found that various drying methods are used during the post-harvest processing of fresh batches of UC and that the quality of the product varies widely between different farmers. The most common drying methods used for the post-harvest treatment of UC are Oven-drying, Sun-drying and Air-drying in the Shade (supporting information Figure S1). The post-harvest processing of raw materials often has an effect on the chemical composition and the bioactivity of the processed products^11^. Therefore, it seems likely that the different drying methods that are traditionally used to process UC may lead to various problems, including an uneven quality of the product. However, research on this issue is scarce, and greater efforts are needed to standardize the post-harvest processing of UC.

Different processing methods cause variations in the chemical constituents and biological activity of plant products, and determining how to chemically and biologically evaluate the effect of the post-harvest treatment is the key to improve the quality control of UC products. First, the variations in the chemical components may include both primary metabolites and secondary metabolites, namely the active compounds. However, most reports fail to consider the whole picture of all metabolites and the potential biotransformation between different metabolites^12^,^13^. Metabolomics is a systematic approach that qualifies and quantifies as many of the metabolites that are present in an organism as possible. NMR-based metabolomics facilitates high-throughput analysis and offers a holistic snapshot of the metabolome, including both primary and secondary metabolites, which is useful in detecting the possible biotransformation between the primary and secondary metabolites during food processing^11^. Second, the changes in the chemical compositions in response to processing methods would lead to changes in biological activity. The beneficial effects of the UC extract cannot be attributed to one single substance, but are believed to involve a number of the constituents found in UC. A biological activity assay analyzes the sum of the all of the components in a plant and the interactions of all components. Therefore, metabolomics combined with biological activity evaluations are applied to explore the effects of different processing treatment on UC and the effects of different drying temperatures.

The aim of this study was to assess the effect of different drying methods on UC herbal tea by (1) evaluating the different post-harvest processing methods used to process UC using metabolomics and biological activity assays and (2) assessing the effect of the Oven-drying temperature on UC quality. Our results show that compared with traditional methods, the Oven-drying methods improve the concentrations of flavonoids and triterpenoids in UC by more than two-fold, accompanied by decreased level of sugars, which indicates that a significant amount of sugars might be biotransformed to flavonoids and triterpenoids during the Oven-drying process. The findings obtained in this study will be useful for the development of novel strategies for post-harvest processing of UC, which, in turn, should enhance the health benefits and save the herbal resource of UC as a functional food.

## Results

### Different chemical profiles of the processed UC samples

#### Metabolomic profiles of the UC samples prepared using the three drying methods

**Figure 1:**
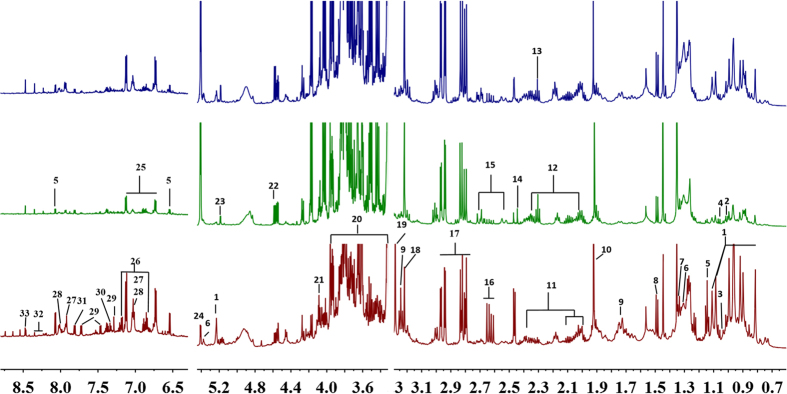
The 600 MHz ^1^H NMR spectra of extracts of the roots of *Uraria crinita* processed by Oven-drying (**A**) Air-drying in the Shade (**B**), and Sun-drying (**C**). The assignments of the metabolites are listed in Table 1. 1, Triterpenoid (with an olean-12-ene skeleton); 2, Leucine; 3, Isoleucine; 4, Valine; 5, Spatholosineside A; 6, Fatty acid; 7, Threonine/Lactate; 8, Alanine; 9, Arginine; 10, Acetate; 11. Proline; 12. Glutamine; 13. γ-Aminobutyric acid (GABA); 14. Succinate; 15. Citrate; 16. Aspartate; 17. Asparagine; 18. Choline; 19. Betaine; 20. Pinitol; 21. Fructose; 22. *β*-Glucose; 23. *α*-Glucose; 24. Sucrose; 25. Unknown 1 (with a *para* —benzene group); 26. Tyrosine; 27. Apigenin 6-C-*β*-D- apiofuranosyl (1→2)-*α*-D-xylopyranoside; 28, Vitexin; 29. Tryptophan; 30. Phenylalanine; 31. Salicylic acid; 32. Adenosine; and 33. Formate.

To investigate the variations in the metabolites present in the UC roots after they were dried using three different post-harvest processing methods, an ^1^H NMR-based metabolomics analysis was used to detect the holistic metabolite profiles of the UC roots. Typical ^1^H NMR spectra of the UC samples processed using the three different drying methods are presented in Fig. 1A; a wide range of metabolites were unambiguously assigned based on the published literature^13^,^14^,^15^,^16^, the database of the Chenomx NMR software suite (Version 7.6, Chenomx, Inc.), and standard compounds. These were confirmed using the JRES, COSY, HSQC, TOCSY and HMBC spectra (Table 1). Thirty-three metabolites were assigned, including amino acids, organic acids, sugars, nucleic acids, amines, triterpenoids, flavonoids and other phenolic compounds (spatholosineside A and salicylic acid), as shown in Fig. 1, Figure S3 and summarized in Table 1, together with their corresponding ^1^H and ^13^C NMR chemical shifts and signal multiplicities. The primary metabolites, particularly amino acids, organic acids and cyclitol (pinitol), were confirmed by GC-MS (Table S1). Snapshots of the three ^1^H NMR spectra indicate that there were significant differences in the phenolic, sugar, organic acid, amino acid, and triterpenoid components when the three different post-harvest processing methods were compared.

#### Multivariate Data Analysis of the UC samples processed by the three drying methods using PCA, PLS-DA, and HCA

**Figure 2:**
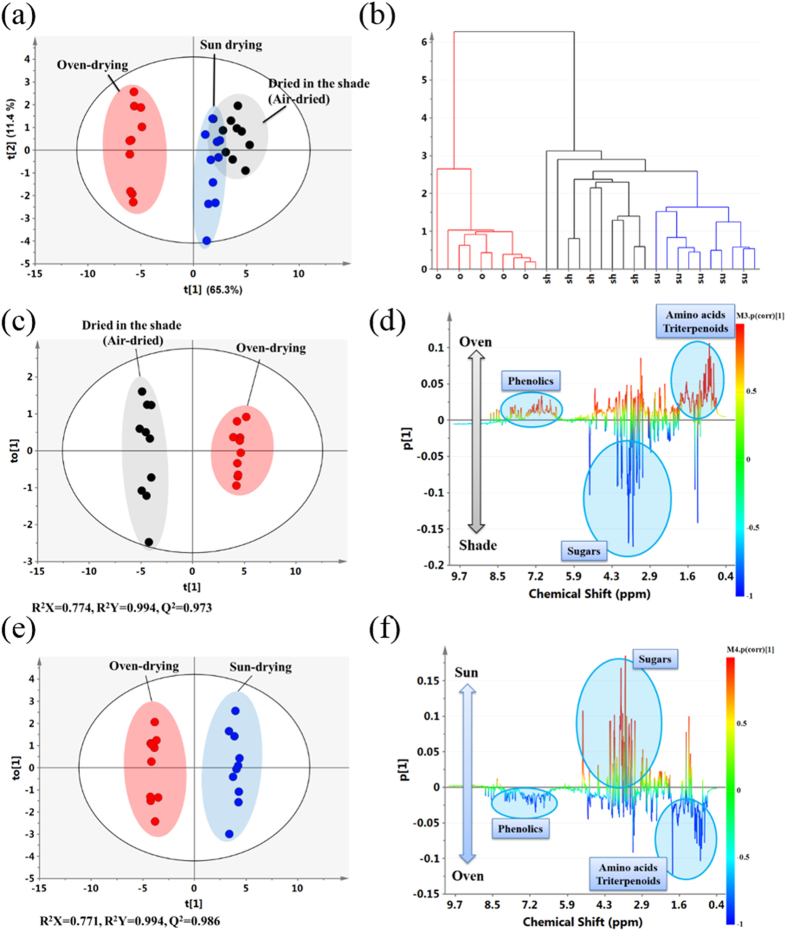
Multivariate data analysis of the ^1^H NMR data obtained from extracts of *Uraria crinita* processed using the three different drying methods. (**A**) PCA score plot. (**B**) Dendrogram plot using HCA. (**C**) Plots of the OPLS-DA scores of the Oven-drying and Shade-drying samples. (**D**) Coefficient-coded loading plots of the Oven-drying and Shade-drying samples. (**E**) Plots of the OPLS-DA scores of the Oven-drying and Sun-drying samples. (**F**) Coefficient-coded loading plots of the Oven-drying and Sun-drying samples.

To comprehensively analyze the information contained in UC extracts from the Sun, Shade and Oven-drying groups, different multivariate data analysis approaches, consisting of PCA, PLS-DA, and HCA, were applied to classify these data. The plots of the PCA scores of the first two principal components with good model quality (PC1 and PC2, R^2^X = 0.759 and Q^2^ = 0.675) show that samples from the Oven group were within a negative region of PC1, whereas the samples in both the Shade group and Sun group were located in a positive region (Fig. 2A), indicating that the samples from the Oven group were clearly distinct from the samples in the Shade group and Sun group. The PLS-DA of the first two components (R^2^X = 0.849 and Q^2^ = 0.966) showed a better separation between the Oven group and the other groups. Furthermore, the classification of the extracts from three drying methods was verified using the HCA dendrogram (Fig. 2B), which showed that the samples in the Oven group were in classified as one group and the samples in both the Shade group and Sun group were classified together in another group.

To identify the metabolites responsible for the difference between the Oven group and Shade/Sun groups, pairwise comparisons were performed between the Oven group and the Shade group, and between the Oven group and the Sun group using PLS-DA and OPLS-DA (Figure S4). The plot of the OPLS-DA scores showed a much clearer separation between the Oven-drying group and the other groups, with higher R^2^ and Q^2^ values than PLS-DA (Fig. 2C). In addition, the OPLS-DA model was validated by CV-ANOVA (Table S2). The corresponding correlation coefficient plot (Fig. 2D) indicated that the extracts obtained from the samples in the Oven group were dominated by a higher level of secondary metabolites, namely triterpenoids with an olean-12-ene skeleton, apigenin glycosides (apigenin 6-C-*β*-D-apiofuranosyl (1→2)-*α*-D-xylopyranoside and vitexin), other phenolics (salicylic acid and unknown 1), spatholosineside A, and higher levels of primary metabolites, namely amino acids (alanine, aspartate, tryptophan, phenylalanine, and tyrosine) and organic acids (formate). There were also lower levels of sugars (glucose and sucrose), citrate compounds and GABA compared with the samples from the Shade group. Similar findings were obtained when the samples from the Oven group and Sun group were compared using this approach (Fig. 2E and F).

To quantify the metabolites that contributed to the differences between the three groups, semi-quantification of some of the important metabolites was performed by integrating the peaks of their typical signals in the spectra (Table 2). Based on our previous study, MCA was used to screen the candidate metabolites to allow us to select the latent metabolites to be studied^17^. It is worth noting that at least two-fold higher concentrations of various secondary metabolites (including apigenin glycosides, spatholosineside A and triterpenoids with an olean-12-ene skeleton) and 5-fold lower concentrations of sucrose were observed in the samples from the Oven group compared with the samples in the other groups (Table 2).

**Figure 3:**
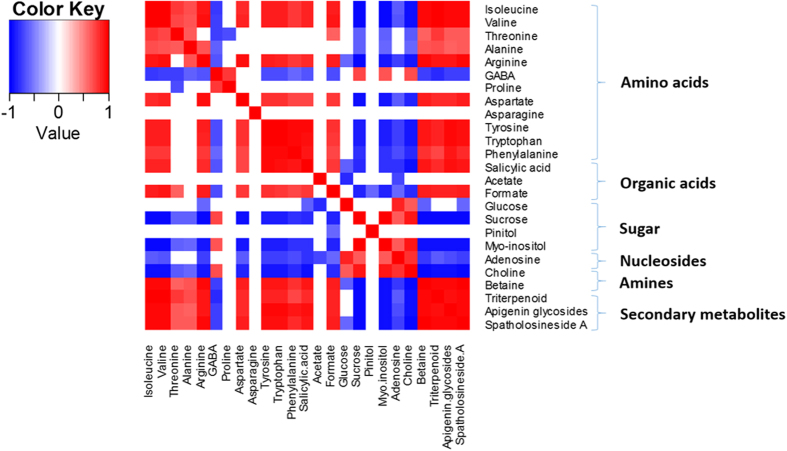
The heatmap of the correlation between the different metabolites in *Uraria crinita* processed with the different drying methods. (Red–positive correlation, Blue–negative correlation).

To determine the relationship between the co-regulated metabolites during processing, a heatmap was constructed based on the correlation coefficients of the metabolites in the different processing groups (Fig. 3). Amino acids showed a positive correlation with organic acids, betaine and the studied secondary metabolites, and a negative relationship with sugars and choline, while sugars showed a negative correlation with amino acids, organic acids and secondary metabolites. A negative correlation was observed between betaine and choline. Thus, the changes in the levels of these metabolites might be related to the biotransformation of the metabolites during the process.

### The effect of temperature on the components present in the oven-dried samples

**Figure 4:**
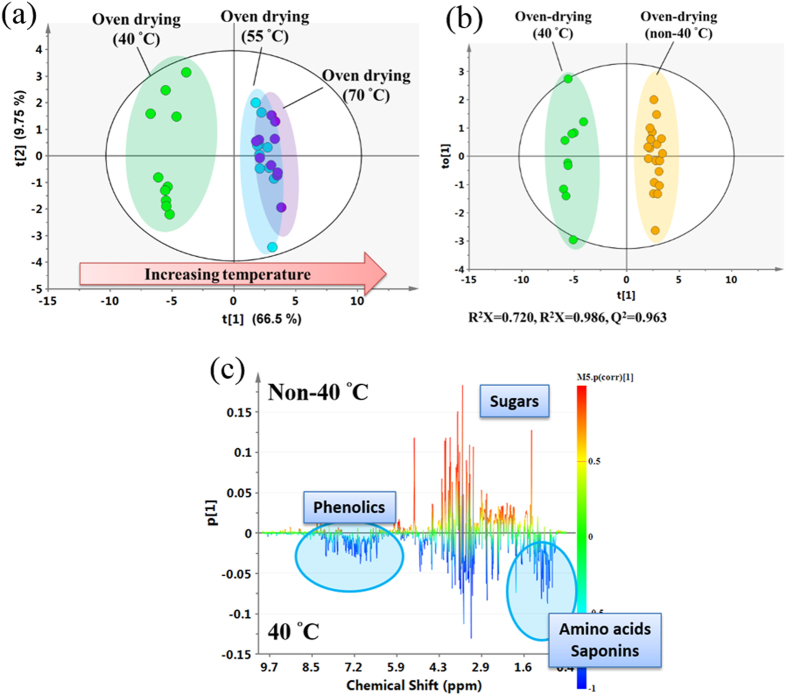
Multivariate data analysis of the ^1^H NMR dataset of extracts of *Uraria crinita* that had been processed by different Oven-drying temperatures at 40 °C and non-40 °C (55 °C and 70 °C). (**A**) Scatter plots of the PCA scores of *Uraria crinita* processed with Oven-drying at 40 °C, 55 °C and 70 °C. (**B**) Scatter plots of the OPLS-DA scores of *Uraria crinita* processed with Oven-drying at 40 °C and non-40 °C (55 °C and 70 °C). (**C**) Coefficient-coded loading plots of the 40 °C and non-40 °C groups.

To evaluate the impact of the temperature used during the Oven-drying method, oven temperatures ranging from 40–70 °C were investigated and detected with ^1^H NMR spectrometry. The ^1^H NMR spectral analysis clearly showed that there were obvious differences between the samples dried at 40 °C and those dried at either 55 °C or 70 °C (Figure S5); these differences included higher levels of triterpenoids, apigenin glycosides, spatholosineside A, other phenolics and amino acids, and a lower level of sugars. Specifically, the plots of the PCA scores demonstrated that the cluster formed by samples in the 40 °C group could be clearly separated from those in the 55 °C group and the 70 °C group (Fig. 4A), which supports the hypothesis that the metabolic characteristics of the 40 °C group are distinctively different from the metabolic characteristics of the 55 °C group and the 70 °C group.

To detect the effects of the 40 °C processing, we divided the samples into two groups, a 40 °C group and a non-40 °C group and analyzed them using the OPLS-DA model (Fig. 4B). Furthermore, the results of the CV-ANOVA validation and perturbation test also show that the OPLS-DA model was not overfitted (Table S3). The plot of the OPLS-DA scores shows a clear separation between the 40 °C and the non-40 °C groups (Fig. 4B). The loading plot of the OPLS-DA model identified the metabolites responsible for the separation (Fig. 4C). Semi-quantification shows that the amounts of the various secondary metabolites present in the samples (triterpenoids with an olean-12-ene skeleton, apigenin 6-C-*β*-D-apiofuranosyl (1→2)-*α*-D-xylopyranoside, vitexin, spatholosineside A and salicylic acid), as well as some primary metabolites (arginine, aspartate, tyrosine, tryptophan, phenylalanine, betaine) were two or more times higher in the samples dried at 40 °C than in the samples dried at the non-40 °C temperatures (Table 3). Taken together, these results suggest that 40 °C is a suitable drying temperature for the UC samples, and higher temperatures cannot increase the levels of the various bioactive metabolites.

**Figure 5:**
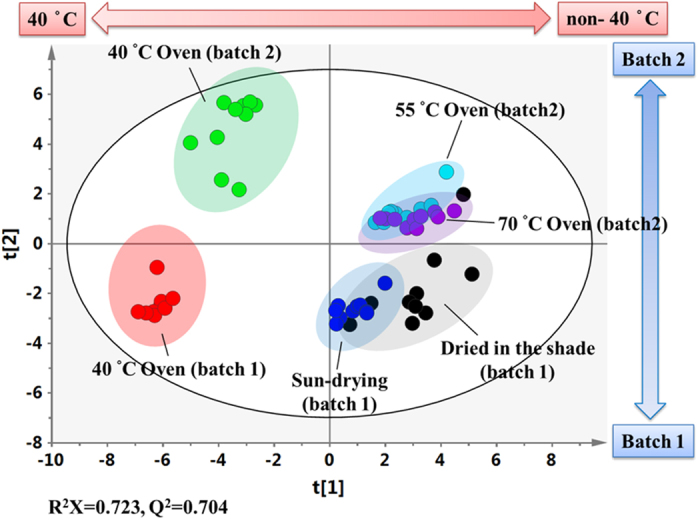
Scatter plots of the PCA scores of the ^1^H NMR dataset of extracts of *Uraria crinita* that had been processed in different batches, by different drying methods, and at different oven temperatures.

To identify the main factors that are responsible for the chemical variations in UC, all of the UC samples from the different processing methods, temperatures and batches were merged together and analyzed by PCA. It is worth noting that all of the samples were clearly separated by PC1 in score plot, with all of the samples at 40 °C in the negative part of PC1 and quite distant from the samples processed at other oven temperatures, whereas all of the samples from the two batches were separated by PC2 (Fig. 5). The separation by PC1 indicates that the chemical composition of UC processed by Oven-drying at 40 °C is quite different from the other samples and the processing temperature is the main factor that influences the chemical composition of UC. In addition, the separation of the two batches in PC2 implies that the collection time can also cause different chemical compositions in UC. However, the temperature-induced differences are far larger than those induced by the collection time, and Oven-drying at 40 degrees will standardize the quality of the UC samples from different batches.

### Variation in the activity of the processed UC samples

**Figure 6:**
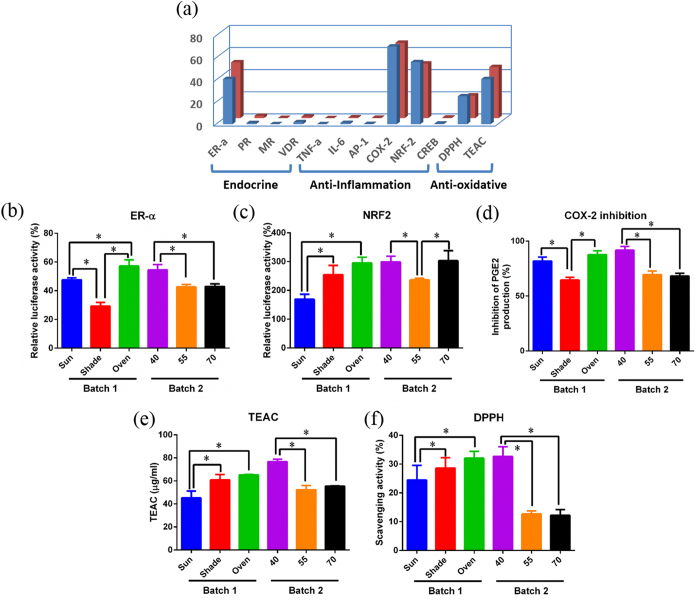
The bioactivity of the extracts of dried roots of *Uraria crinita* from different batches that had been processed by different drying methods at different oven temperatures. (**A**) The overall screening test; (**B**) Estrogen receptor-α (ER-alpha); (**C**) Nuclear factor erythroid-2-related factor (Nrf2); (**D**) COX-2; (**E**) DPPH; (**F**) Trolox equivalent antioxidant capacity (TEAC) assay.

The bioactivity of UC sample was screened in different cell lines by analyzing the levels of endocrine receptors activities (ER-α, PR, MR, and VDT), anti-inflammatory signaling activities (TNF-alpha, IL-6, AP-1, and Nrf2) and enzyme activity (COX-2), and by performing an anti-oxidative assay with DPPH and TEAC. The result showed that the UC sample significantly stimulates ER-α and Nrf2 signaling activities, significantly inhibits the ability of COX-2 to generate PGE2, and a good anti-oxidative effect in DPPH and TEAC assays (Fig. 6A). Thus, we performed the five effective assays (ER-α, Nrf2, COX-2, DPPH and TEAC) to evaluate the effect of the three different post-harvest treatments and the different Oven-drying temperatures on the biological activity of the UC samples. Figure 6 showed that the Oven group exhibited significantly higher activities than those of the Shade group and the Sun group. These results suggested that Oven-drying at 40 °C is likely to have a positive effect on the bioactivity of UC.

## Discussion

### The correlation between the chemical components, bioactivities and the traditional usage of UC

This study performed an evaluation of UC quality after post-harvest Oven-drying processing by combining metabolomics with bioactivity assessments. Our results determined that the UC extracts stimulateER-α receptor and Nrf2 receptor and increase the anti-oxidation activity of HEK293 cells (Fig. 6). The UC extract is dominated by amino acids, organic acids, sugars, flavonoids and terpenoids.

Estrogen regulates the skeletal system. Decreased estrogen levels will induce T cell proliferation, up-regulate TNF-α, promote osteoclast activation, and result in postmenopausal osteoporosis^18^. Our study has found that UC has a fairly good effect as a phytoestrogen; this might be correlated with the flavonoid levels in UC^19^. This result is consistent with previous studies showing that the flavonoids in UC can stimulate the osteogenic activity of osteoblasts^19^,^20^. Moreover, we identified another active ingredient, pinitol, which can inhibit the formation of osteoclastogenesis^21^. Generally, UC regulates bone metabolism, and this discovery has a close relationship with its traditional use to improve children’s development and in traumatic injury medicine.

The Nrf2/ARE pathway regulates the expression of anti-oxidative proteins, which protect against the damage caused by injury or oxidative stress resulting from inflammation^22^. The Nrf2/ARE pathway also regulates the expression of phase II detoxifying enzymes and transporters^23^. Our study shows that UC stimulates Nrf2 (Fig. 6) and exhibits a free radical-scavenging effect. This result is support by a previous finding that UC has anti-oxidative and nitric oxide-scavenging activities^9^. Additional studies are still required to confirm whether the anti-inflammation activity of UC is mediated by regulating the Nrf2 pathway. The UC-mediated regulation of Nrf2 and anti-oxidation effects might be correlated with the levels of flavonoids, triterpene and betaine, while the COX-2 enzyme activity and anti-inflammation effects may be related to flavonoids and salicylic acid^24^,^25^. These activities of UC may be relevant to the traditional uses of UC: detoxification, detumescence and the treatment of traumatic injuries.

The combination of NMR-based metabolomics and GC-MS profiling led to the discovery of pinitol in the UC used in our studies, which is the first report of pinitol in UC. Pinitol has anti-diabetes effects and improves metabolic disease, which was confirmed by clinical data and basic research^24^,^25^,^26^,^27^,^28^. This discovery indicates the potential, novel use of UC to treat diabetes. Concerning the wide use of UC in folk medicine, UC might be developed as a treatment for metabolic diseases in the future, which may expand the utilization of this medicinal plant.

### The improvement in the levels of the bioactive compounds would seem to be beneficial to the quality of functional foods or teas

Large variations in the concentrations of the bioactive compounds present in herbal teas have been demonstrated in many reports and this has created great concern in terms of its effect on the quality and bioactivity of herbal tea or functional foods^12^. This variation has been attributed to a variety of different agricultural factors, including the environment, climate, collection time and growing year^12^. However, in general, the chemical variations associated with the post-harvest drying methods have been overlooked in these studies. One important discovery of the present study is that the different traditional drying methods greatly affect the concentrations and bioactivities of the active ingredients in UC. Oven-drying at 40 °C greatly increases the levels of triterpenoids and apigenin glycosides (Table 2), and accordingly improves its bioactivities on the ER-α receptor and Nrf2 receptor, as well as its anti-oxidation effects (Fig. 6). Furthermore, we have also found that the temperature in the drying process is a key factor that has a significant effect on the concentrations and bioactivities of the active components present in the dried products. A comparison of the whole chemical profiles and the levels of some bioactive components (triterpenoids, flavones, and spatholosineside A) in the samples dried at 40 °C, 55 °C and 70 °C reached the same conclusion, namely that the Oven-drying temperature had a significant effect on the levels of the active components in the UC samples (Fig. 4 and Table 3). This conclusion is also confirmed by the increased ER-α receptor and antioxidative activities of the sample dried at 40 °C compared with the other temperatures (Fig. 6). Therefore, the drying methods and temperature used in the drying process play important roles, and the concentrations of the bioactive components can be improved by Oven-drying methods at an appropriate temperature.

Methods that improve the consistency of the quality of herbal tea or functional foods is an important to improve the consistency of their clinical curative effects. The cost of the traditional, natural drying methods is low, but changing weather conditions greatly influence the quality of the products, leading to poor uniformity in the quality of the products. The chemical composition is the basis of bioactivity, and increasing the consistency of the chemical composition further increases the consistency of efficacy. Compared with other drying methods, the Oven-drying method is costlier, but it reduces the impact of climate factors on the quality of the product and ensures the consistent product quality. Our data show that a cluster of the samples obtained by Oven-drying at 40 °C across two batches of UC were in the negative part of PC1 in the score plot (Fig. 5) and this is a quite distant from the samples processed at the other oven temperatures. In addition, the two batches can be separated by PC2 of the PCA plot in Fig. 5, this variation is mainly due to the differences in collection time, because all of the samples from batch one were located in the negative region of PC2, while those from batch two were in the positive region of PC2. The levels of some metabolites, such as pinitol, might be correlated with the collection time (Tables 2 and 3), because there was a difference in their concentrations between the different batches, but no difference between the drying temperatures and methods. These data indicated that the quality of the UC samples that were oven-dried at 40 °C is distinctively different from those prepared at other temperatures and by other processing methods. This is also confirmed by the higher bioactivities of the UC samples from the two batches that were oven-dried 40 °C compared with the other samples (Fig. 6). However, whether the application of Oven-drying at 40 °C results in good reproducibility across the UC samples are still needed more experiments with samples from different fields, areas or years.

### Biotransformation in different biopathways might be involved in the drying process

**Figure 7:**
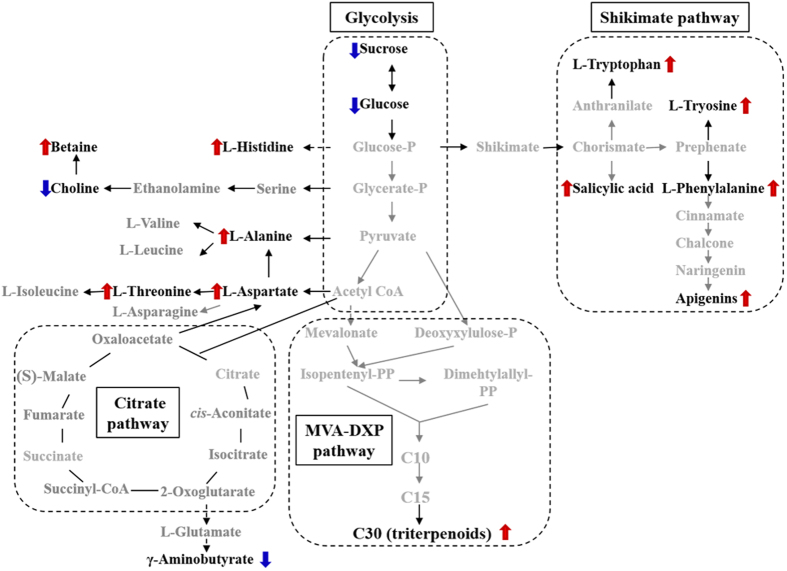
The proposed alterations in the metabolic pathway and an outline of some of the metabolites of *Uraria crinita* that are affected by Oven-drying at 40 °C compared with Sun-drying and Air-drying in the shade.

Traditionally, it was thought that Sun-drying or drying at high temperature might increase the hydrolysis of glycosides to aglycones, particularly for flavonoids. Interestingly, we found that Oven-drying at 40 °C increased the contents of flavonoids, triterpenoids, amino acid, and betaine, accompanied by a decrease in the amount of sugar (Fig. 1 and Table 2), suggesting that biotransformation might occur during processing. Furthermore, the temperature is a key factor for this biotransformation, as the levels of these secondary metabolites decreased when the processing temperature increased from 40 °C to 70 °C (Fig. 4). These results imply that post-harvest Oven-drying processing at 40 °C might improve the biotransformation of sugars to secondary metabolites. From the perspective of plant physiology, freshly harvested plant materials, particularly the roots, are physiologically active. As a result, water depletion during the drying process will cause a series of dehydration-related physiological reactions that maintain the normal physiological function^29^,^30^,^31^,^32^, particularly the increase in the levels of some secondary metabolites that are involved in defense mechanisms^33^. The increase in the levels of betaine and free amino acids in plants as a result of drought stress can regulate the osmotic balance to maintain the ability to resist drought^30^,^31^; the biosyntheses of betaine, flavonoids and triterpenoids improve the plants’ resistance to the increased oxidative stress caused by drought stress, thus protecting the plant cells and maintaining normal function^29^,^32^,^34^. Salicylic acid can regulate the expression of anti-oxidase to improve the plants’ resistance by changing active oxygen metabolism and associated signal transduction pathways^35^. Three main metabolic pathways might be involved in the biosynthesis of the active ingredients in UC during post-harvest processing (Fig. 7): 1) glycolysis and the shikimate pathway; 2) the biosynthesis of betaine; and 3) the biosynthesis of triterpenoid.

The shikimate pathway is the main hub that connects glycolysis and secondary metabolism in higher plants, fungi and bacteria. After chorismate is formed, aromatic amino acids (tryptophan, tyrosine and phenylalanine)^36^,^37^ and salicylic acid are then synthesized, eventually leading to flavonoid synthesis. The up-regulation of these metabolites plays an important role in plant physiology and resistance to environmental stresses^36^,^37^. Compared with the other groups, the aromatic amino acid (aromatic amino acids), salicylic acid and flavonoid concentrations were significantly increased in the Oven-drying group, accompanied by down-regulation of large amounts of carbohydrates. It indicates that biosynthesis through the shikimate pathway is likely to be major mechanism of the biotransformation reactions produced at 40 °C, resulting in massive transformation of the metabolites from sugars to flavonoids.

The biosynthesis of triterpenoids from sugars occurs via the mevalonic acid-deoxyxylulose phosphate (MVA-DXP) pathway in plants^38^,^39^,^40^. The increased triterpenoid levels are also related to the resistance to environmental stress^32^. From a metabolomics perspective, it is obvious that drought stress caused by drying induces a higher level of triterpenoids in the Oven group compared with the other groups. Drought stress in *Glycyrrhiza uralensis*, a legume plant, was also reported to up-regulate the expression of the triterpene synthase gene and increase the level of glycyrrhizic acid^32^. This report supports our current finding, but further studies are still needed to verify the mechanism.

The presence of betaine is correlated with resistance to osmotic stress in plants^33^. It is a non-toxic agent that protects against protein penetration and can reduce lipid peroxidation in the plant. It is well known that choline is the precursor of betaine^33^. The decreased level of choline and increased level of betaine indicate that the biosynthesis of betaine from choline occurs in UC during processing at 40 °C.

Taken together, the results of this study proposed that post-harvest drying processing induces the important biotransformation of metabolites through multiple metabolic pathways, such as glycolysis, the shikimate pathway and the MVA-DXP pathway. These biotransformations involve the biosynthesis of aromatic amino acids, salicylic acid, flavonoids, triterpenoids and betaine in response to dehydration. Therefore, a drying method that uses an appropriate temperature can promote the quality of UC, with regard to both the levels of the active components and their biological activities.

## Conclusions

This study used metabolomics combined with a biological activity analysis to explore the significance of the post-harvest processing of UC. This investigation demonstrates that Oven-drying at 40 °C increases the accumulation of active components and the biological activity of UC. In terms of their efficacy and consistency, the quality of the UC samples can be significantly improved by the use of a postharvest Oven-drying method. It is worth generalizing this method to the post-harvest of UC. Overall, the present study sheds light on methods to improve the quality of the raw materials used as an herbal tea ingredient by adjusting their post-harvest Oven-drying method.

The NMR-based metabolomics approach applied in this study provides a full picture of both the primary and secondary metabolites in the UC samples, which facilitates the interpretation of biotransformations between primary and secondary metabolites. NMR-based metabolomics helps us to understand the possible biotransformations of the primary metabolites to the secondary metabolites that may be occurring when the UC roots are subjected to the drying process.

## Materials and Methods

### Chemicals

D-methanol (99.9%), D_2_O (99.9%), 1,1-diphenyl-2-picrylhydrazyl (DPPH), 2,2″-azinobis (3-ethylbenzothiazoline-6-sulfonic acid) diammonium salt (ABTS), 6-hydroxy-2,5,7,8- tetramethylchromane-2-carboxylic acid (Trolox), gallic acid, pinitol, quercetin, methoxyamine-HCl, N-methyl-N-(trimethylsilyl) trifluoroacetamide (MSTFA), pyridine, and potassium dihydrogen phosphate (KH_2_PO_4_) were purchased from Sigma-Aldrich (St. Louis, MO). Trimethylsilane propionic acid sodium salt (TSP) was obtained from Merck (Darmstadt, Germany). Sodium deuteroxide (NaOD) was purchased from Cambridge Isotope Laboratories (Tewksbury, MA). Coenzyme-A lithium was purchased from United States Biological (Salem, MA). Potassium luciferin was purchased from Gold BioTechnology (St. Louis, MO). The COX Inhibitor Screening Assay Kit (560131) was purchased from Cayman Chemical Company. The HEK293 cell lines transfected with the pER-Luc vector to express the estrogen-alpha receptor and a reporter gene vector or the pNrf2-Luc vector to express Nrf2 and a reporter gene were obtained from Dr. Yung-Chi Cheng’s lab.

### Sample collection and the various drying processes

The roots of UC were collected from Nantou County, Taiwan and were identified by the authors using various molecular approaches (Figure S2). The cultured UC was collected during October (first batch) and November (second batch) in the morning, which coincides with the traditional fall collection season of farmers^3^,^5^. Plant voucher specimens (LMS-1001) were deposited at the Department of Chinese Pharmaceutical Sciences and Chinese Medicine Resources of the China Medical University. All samples were confirmed by molecular identification.

The two different batches of UC roots were separately and rapidly washed. The roots of the first batch were divided into three different groups, including 1) the Oven group, where the plant material was dried in an oven for two days at 40 °C; 2) the Shade group, where the plant material was dried in the Shade (Air-drying) for 12–14 days outside at ambient temperatures ranging from 22–25 °C; and 3) the Sun group, where the plant materials were dried in the Sun for 5–7 days at ambient temperatures ranging from 22–28 °C. The plant material from the second batch was separated into three groups and dried in an oven at different temperatures, namely 40 °C, 55 °C and 70 °C, for two days. All of the samples from the different plant material groups were dried to a constant weight to exclude the effects of moisture during the biological activity assays.

### Cell culture

The experiment was performed in the laboratory of Dr. Yung-Chi Cheng’s lab of Yale University in the United States. The human embryonic kidney (HEK) 293 cells were purchased from the American Type Culture Collection (Manassas, VA). The HEK-293 cells were grown in DMEM medium (Dulbecco’s Modified Eagle’s Medium) (GIBCO 11965 base, Life Technologies, Carlsbad, CA) containing 10% fetal bovine serum (FBS) and 100 μg/mL kanamycin in a humidified CO_2_ (5%) incubator at 37 °C. Before seeding, the pER-α-Luc HEK-293 cells were washed with PBS and cultured in DMEM medium (GIBCO 21063) containing 5% charcoal dextran-treated FBS (cdFBS) for 2 days to starve the cells. The PER-α-Luc HEK-293 cells were seeded onto 96-well plates at 40,000 cells/well in 0.1 mL of estrogen-free medium. The HEK-293 pNrf2-Luc cells were inoculated onto a 996-well plate at 20,000 cells/well in 0.1 mL of DMEM containing 10% FBS.

### Biological activity tests

#### Anti-oxidative assay

The UC roots from the different treatment groups were ground with a mortar and pestle and then extracted with 500 mL of 50% ethanol three times over 7 days. The extracts were then filtered, combined and evaporated with a rotary evaporator at 40 °C. The DPPH radical scavenging assay was performed as previously described^41^,^42^, with slight modifications. The UC extracts from the different groups (1 mg/mL, 0.1 mL) were added to a methanolic solution (0.1 mL) of DPPH radical (the final concentration of DPPH was 0.2 mmol/L). The mixture was shaken vigorously in the dark and then incubated for 30 min at room temperature. The absorbance of the resulting solution was measured spectrophotometrically at 517 nm, and the results were used to calculate the scavenging effect of each UC sample using the equation:


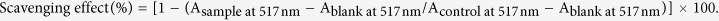


The Trolox equivalent antioxidant capacity (TEAC) assay was performed to test the total antioxidant levels, as previously described^41^,^42^,^43^. The UC extract solution (1 mg/mL) or Trolox solution were mixed with 0.125 mL of ABTS (1000 μM), 0.125 mL of H_2_O_2_ (500 μM) and 0.125 mL of peroxidase (44 unit/mL). The mixture was incubated for one hour in the dark until ABTS^+^ radical cation was generated. After incubation, the absorbance of the solution was measured at 734 nm over 10 minutes. The Trolox dose-response (curve) curve was constructed, and the antioxidant ability was expressed in TEAC.

#### COX-2 enzyme inhibitor screening assay

The test was performed using the protocol provided with the COX Inhibitor Screening Assay Kit (560131) from Cayman Chemical Company.

#### ER-α promoter assay in HEK293 cells

An appropriate concentration of the UC extract was added to the pER-α- Luc HEK-293 cells and incubated for 24 h. 17-β -Estradiol (Sigma) was dissolved in dimethyl sulfoxide and used as a positive control. Then, the medium was aspirated, the cells were lysed in 0.5 mL/well of lysis buffer (1% Triton X-100, 2 mM dithiothreitol, 2 mM trans-1,2-Diaminocyclohexane-N,N,N′,N′-tetraacetic acid monohydrate, 10% glycerol and 25 mM Tris-HCl, pH 7.8) for 10 min, and 0.1 mL/well of luciferase assay buffer (20 mM Tris-HCl, pH 7.8, 10 mM NaHCO_3_, 2.5 mM MgSO_4_ 0.1 mM EDTA, 10 mM DTT, 60 μM coenzyme-A lithium, 225 μM potassium luciferin and 250 μM ATP) was added, and the luciferase activity was determined immediately and calculated using the following equation:


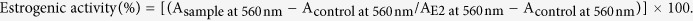


#### Nuclear factor erythroid-2-related factor (Nrf2) promoter assay in HEK293 cells

An appropriate concentration of the UC extract was added to the pNrf2-Luc HEK 293 cells and incubated for 16 h before the levels of Nrf2 were determined. Andrographolide (20 μM) dissolved in dimethyl sulfoxide was used as a positive control. The other steps are the same as the methods used in 2.4.3.

### NMR metabolomics

#### Sample preparation

The samples were prepared for the NMR metabolomics analysis using a previously described procedure^16^ with slight modifications. The powdered material (50 mg) was transferred to an Eppendorf tube; then, 0.75 mL of CH_3_OH-*d*_4_ and 0.75 mL of KH_2_PO_4_ phosphate buffer (90 mM, pH 6.0, containing 0.01% TSP in D_2_O; the pH was adjusted using a 1 M NaOD solution) were added to the tube. The tube was vortexed for 1 min at room temperature, followed by ultrasonication for 30 min at 298 K; the sample was then centrifuged at 13,200 rpm for 10 min at 298 K. Finally, 600 μl of the supernatant were transferred to a 5-mm NMR tube for NMR analysis.

#### NMR measurements

The samples were analyzed on an AVANCE AV-600 MHz spectrometer with a cryogenic probe as previously described^14^,^15^, with slight modifications. All ^1^H NMR spectra were recorded at 298 K. Manual shimming was performed on each sample to reach a full width at half maximum (FWHM) ≦ 1.8 Hz on the TSP peak of each sample (using the Bruker “zggppr” pulse sequence and a line broadening of 0.3 Hz). The ^1^H-NMR spectra were acquired using a standard one-pulse sequence with water saturation (zggppr) and the following parameters: number of scans (NS) = 128, number of dummy scans (DS) = 0, time domain (TD) = 32 K data points, spectral width = 20 ppm, relaxation delay (RD) = 2.0 sec, and acquisition time = 1.36 sec. The 90° pulse length (~10.5 μs) was individually adjusted for each sample. The free induction decays (FIDs) were Fourier transformed using LB = 0.3, and were zero-filled to produce 65,536 points.

For spectral resonance assignment purposes, ^1^H-^1^H correlation spectroscopy (COSY), ^1^H-^1^H total correlation spectroscopy (TOCSY), ^1^H J-resolved spectroscopy (JRES), ^1^H-^13^C heteronuclear single-quantum coherence (HSQC) and heteronuclear multiple bond correlation (HMBC) 2D NMR spectra were acquired on selected samples and processed as previously reported^14^,^15^.

### Data processing and Multivariate Data Analysis

The resulting ^1^H-NMR spectra were manually phased, baseline corrected, and calibrated to a TSP at δ = 0.00 ppm using Mestrenova software (version 8.0.2, Mestrelab research S.L.). The spectral range of δ 0.04–10.00 was bucketed into 1,992 bins (0.005 ppm), with the regions from δ 3.300–3.400 and δ 4.700–5.000 excluded. The integral values of spectrum segments were normalized to a total integrated area of the spectrum to reduce the differences in concentrations between samples^17^.

The resulting NMR datasets was imported into SIMCA-P version 13.0 (Umetrics, Umea, Sweden). All variables were scaled to Pareto (par) for multivariate statistical analyses (principal components analysis (PCA), partial least squares discriminant analysis (PLS-DA), hierarchical cluster analysis (HCA), and orthogonal partial least squares discriminant analysis (OPLS-DA)). The quality of the fitting model can be explained by the appropriate R^2^ and Q^2^ values. R^2^ is defined as the total amount of variation explained by the model and Q^2^ is the indicated predictability of the model under cross validation^17^,^44^. The PLS-DA was validated by the permutation test (200 permutations)^17^,^45^. OPLS-DA was validated by both a 7-fold cross-validation and ANOVA of the cross-validated residuals (CV-ANOVA)^45^,^46^. A multi-criteria assessment (MCA), including the coefficient values, variable importance in projection (VIP) values and *p* values, were used to screen and select the latent metabolites^17^. The MCA was performed using the followed criteria: 1. coefficient value |r| > 0.576; and 2. VIP > 1, and 3. *p* value < 0.05^17^. The heat map was constructed using R software 3.2.1 (R-Foundation for statistical computing, www.Rproject.org).

All of the results are presented as means ± SE. The statistical analysis was performed using one-way ANOVA, followed by Bonferroni’s *post hoc* test. The criterion used for statistical significance was *p* < 0.05.

## Additional Information

**How to cite this article**: Chao, J. *et al*. Improving the Concentrations of the Active Components in the Herbal Tea Ingredient, *Uraria crinita*: The Effect of Post-harvest Oven-drying Processing. *Sci. Rep.* **7**, 38763; doi: 10.1038/srep38763 (2017).

**Publisher's note:** Springer Nature remains neutral with regard to jurisdictional claims in published maps and institutional affiliations.

## Supplementary Material

Supplementary Data

## Figures and Tables

**Table 1:** Assignment of the NMR signals for the *Uraria crinita* samples.

	Metabolites	Assignment	^1^H (multiplicity), coupling constant, multiplicity	^13^C	References
1	Triterpenoid (with olean-12-ene skeleton)	Six angular methyl groups	0.81^※^, 0.89, 0.92, 0.96,0.99,1.08,1.11 (s)	19.6, 16.6, 16.0, 32.1, 17.01, 28.9, 32.1	COSY, JRE, HSQC, HMBC
		C_12_-H	5.23 (br)	123.5	
2	Leucine	β-CH_2_	1.72 (m)	46.5	Database, COSY, HSQC
		δ-CH_3_	0.98	22.4	
3	Isoleucine	γ-CH_3_	1.03 (d, *J* = 7.0 Hz)^※^	17.9	Database, COSY, HSQC, JRE, GC-MS
		δ-CH_3_	0.98 (t)	14.1	
4	Valine	α-CH_2_	3.60	56.7	Database, COSY, HSQC, JRE
		γ-CH_3_	1.01 (d, *J* = 6.2)	19.14	
		γ‘-CH_3_	1.06 (d, *J* = 6.2)	24.57	
5	Spatholosineside A	C_5_-H	8.07 (d, *J* = 6.0 Hz)*	157	Mao, *et al*.^19^; COSY, JRE, HSQC, HMBC
		C_6_-H	6.54 (d, *J* = 6.0 Hz)	116.69	
		β-Glc-1H	4.89(d, *J* = 7.5 Hz)	103	
		α-Rhm-1H	4.74(br)	101	
		α-Rhm-6H	1.14(d, *J* = 6.0 Hz)	17	
		CH_3_	2.46 (s)	16.0	
6	Fatty acid	CH_3_	0.87	16.4	Fatty acid
		CH_2_	1.30	29.7	
		Double bound	5.38 (m)	130	
		=CCH	2.04	23	
7	Threonine/Lactate	β-CH	4.28	69.1	Database, COSY, HSQC, JRE
		γ-CH_3_	1.34 (d, *J* = 6.6 Hz)	22.5	
8	Alanine	α-CH	3.71 (d, *J* = 7.2)	53.8	Database, COSY, HSQC, JRE
		β-CH_3_	1.49 (d, J = 7.2),	17.15	
9	Arginine	β-CH_2_	1.9 (m)	26.3	Database, COSY, HSQC, JRE
		γ -CH_2_	1.67,1.71 (m)	24.1	
		δ-CH_3_	3.24 (t)*	41.96	
10	Acetate	CH_3_	1.92 (s)*	24.3	Database, HSQC, HMBC
11	Proline	α-CH	4.08 (m)	64.1	Database, COSY, HSQC
		β-CH_2_	2.32–2.38 (m)	30.0	
		γ -CH_2_	1.95–2.11 (m)	25.2	
12	Glutamine	α-CH	3.71	55.39	Database, COSY, HSQC
		β-CH_2_	2.15 (m)	29.68	
		γ -CH_2_	2.46 (m)	34.48	
		α′-CH	2.70 (d)		
13	γ-Aminobutyric acid (GABA)	α-CH_2_	2.30 (t)*	37.3	Database, COSY, HSQC, JRE
		β-CH_2_	1.89 (m)	26.8	
		γ -CH_2_	3.01 (t)	42.1	
14	Succinate	CH_2_	2.44 (s)	35.8	Database, HSQC
15	Citrate	α-CH	2.54 (d)	48.6	Database, COSY, JRE HSQC
		α′-CH	2.70 (d)		
16	Aspartate	β-CH	2.63 (dd)*	37.53	Database, COSY, HSQC, JRE
		β-CH	2.81 (dd)	35.20	
17	Asparagine	β-CH	2.81 (dd)	35.20	Database, COSY, HSQC, JRE
		β′-CH	2.95 (dd)*	35.56	
18	Choline	N-CH_3_	3.21 (s)^※^	54.3	Database, COSY, HSQC
		N-CH_2_	4.05	70.3	
		CH_2_OH	3.55	56.2	
19	Betaine	NCH_3_	3.28 (s)*	53.4	Database, COSY, HSQC
		CH_2_OH	3.91 (s)	66.7	
20	Pinitol	6-OCH_3_	3.61 (s)^※^	62.2	Database, COSY, HSQC, TOCSY, GC-MS
		CH-OH	3.33	85.5	
		CH-OH	3.64	74.8	
		CH-OH	3.72	73.4	
		CH-OH	3.78	72.6	
		CH-OH	3.96 (t)	74.4	
21	Fructose	C3-H	4.08	82.9	Database, COSY, HSQC, JRE,
		C4-H	3.96 (br)	77	
		C5-H	4.03	82.2	
22	β-Glucose	β-C1H	4.58 (d, *J* = 7.9)	97.03	Database, COSY, HSQC, JRE
23	α-Glucose	α-C1H	5.19 (d, *J* = 3.8)*	92.7	
24	Sucrose	Glc-C1H	5.41 (d, *J* = 3.9) *	92.6	Database, COSY, HSQC, JRE
		Fru-C3H	4.17 (d, *J* = 8.6)	77.69	
25	U1 (*para* —benzene)		6.73(d, *J* =8.7 Hz)	103	HSQC, HMBC
			7.12(d, *J* =8.7 Hz)	132	
				155	
26	Tyrosine	C2,6 -H	7.18 (d, *J* = 8.6)	131.29	Database, COSY, HSQC, JRE
		C3,5 -H	6.84 (d, *J* = 8.6)*	117	
		β-CH	3.02 (dd, *J* = 15, 8.1)	42.3	
		β′-CH	3.20 (dd, *J* = 15, 4.4)	42.3	
27	Apigenin 6-C-β-D- apiofuranosyl(1→2)-α-D-xylopyranoside	C3-H	6.80 (s)	103	Mao, *et al*.^19^; COSY, JRE, HSQC, HMBC
		C8-H	6.65 (s)	94	
		C2′-H, C6′-H	7.92 (d, *J* = 8.6 Hz)	129.8	
		C3′-H, C5′-H	7.02 (d, *J* = 8.6 Hz)^※^	116.7	
		β-Xyl-C1-H	4.55 (br, s)	73.7	
		α-Api-C1-H	5.15 (br, s)	108	
28	Vitexin	C3-H	6.60 (s)	105.6	Mao, *et al*.^19^; COSY, JRE, HSQC, HMBC
		C6-H	6.39 (s)	99	
		C2′-H, C6′-H	8.01 (d, *J* = 8.6)	129.6	
		C3′-H, C5′-H	7.02 (d, *J* = 8.6)^※^	117.2	
		Glc-C1-H	4.55 (d, *J* = 8.3)	73.8	
29	Tryptophan	C2-H	7.29 (s)*	124.9	Database, COSY, HSQC, JRE
		C4-H	7.72 (d, *J* = 7.6)	119	
		C5-H	7.14 (t, *J* = 7.6)	122.5	
		C6-H	7.22 (t, *J* = 7.6)	119.7	
		C7-H	7.46 (d, *J* = 7.6)	112.3	
30	Phenylalanine	C2,6-H	7.34 (m)	129.3	Database, COSY, HSQC
		C3,5-H	7.41 (m)	129.1	
		C4-H	7.39 (m)	133.1	
31	Salicylic acid	C3-H	6.75 (d, *J* = 8,1)	115.5	Mao, *et al*.^19^; Database, COSY, HSQC, JRE
		C4-H	7.38 (t, *J* = 8.1)	135.1	
		C5-H	6.90 (t, *J* = 8.1)	119.6	
		C6-H	7.8 (d, *J* = 8.1)*	131.0	
32	Adenosine	C1′-H	6.02 (d, *J* = 6.6)	89.7	Database, HSQC, JRE
		C2-H	8.35 (s)^s※^	152.7	
		C8-H	8.23 (s)	142.8	
33	Formate	HC=O	8.47 (s)	—	Database

**Table 2:** Normalized integral values of the metabolites from the NMR spectra across the extracts from *Uraria crinita* that were processed by the three different drying methods.

Metabolites	Normalized integral value (NMR signal × 10^3^)^a^		
	Sun	Shade	Oven
**Amino acids**			
Threonine/Lactate	415.73 ± 27.66^c^	385.51 ± 40.08^b^	457.86 ± 30.77b,c
Arginine	621.33 ± 58.22^c^	560.45 ± 100.78^b^	1113.02 ± 54.82b,c
γ-Aminobutyric acid (GABA)	549.74 ± 36.83c,d	620.03 ± 83.73b,d	440.37 ± 48.5b,c
Aspartate	572.5 ± 73.51^c^	485.34 ± 158.61^b^	926.74 ± 90.1b,c
Asparagine	2121.02 ± 77.78	2056.79 ± 191.38	2033.91 ± 182.48
Tyrosine	14.51 ± 4.47^c^	9.83 ± 4.69^b^	88.78 ± 25.17b,c
Tryptophan	—	—	44.91 ± 14.32
Phenylalanine	—	—	22.99 ± 9.34
**Organic acids**			
Salicylic acid	16.17 ± 1.04^c^	20.6 ± 2.15^b^	43.13 ± 7.65b,c
Acetate	519.17 ± 40.46c,d	894.45 ± 89.02^d^	948.17 ± 51.51^c^
Formate	13.55 ± 2.06^c^	10.67 ± 1.95^b^	23.75 ± 4.00b,c
**Sugars and sugar alcohols**			
Glucose	102.87 ± 11.86c,d	66.56 ± 13.39b,d	52.32 ± 10.15b,c
Sucrose	313.26 ± 8.78c,d	409.98 ± 20.35b,d	52.55 ± 1.71b,c
Pinitol	2314.26 ± 136.45	2525.75 ± 389.88^b^	2089.93 ± 116.36^b^
**Others**			
Adenosine	12.69 ± 1.06c,d	8.62 ± 1.19b,d	5.14 ± 0.73b,c
Choline	949.35 ± 48.34^c^	948.27 ± 40.55^b^	596.77 ± 34.31b,c
Betaine	141.12 ± 18.1^c^	112.61 ± 26.54^b^	712.17 ± 28.67b,c
**Secondary metabolites**			
Triterpenoid (with olean-12-ene skeleton)	388.43 ± 44.28c,d	292.6 ± 52.05b,d	849.1 ± 62.01b,c
Apigenin glycosides (27+28)	142.23 ± 19.87^c^	104.63 ± 29.64^b^	364.54 ± 50.05b,c
Spatholosineside A	20.86 ± 2.33c,d	16.67 ± 2.92b,d	58.89 ± 4.41b,c

^a^The results are shown as means ± SE. The statistical analysis was performed using the independent sample *t*-test and one-way ANOVA, followed by Bonferroni’s *post hoc* test. The criterion used for statistical significance was *p* < 0.05.

^b^*p* < 0.05, Shade versus Oven.

^c^*p* < 0.05, Sun versus Oven.

^d^*p* < 0.05, Sun versus Shade.

**Table 3:** Normalized integral values of the metabolites from the NMR spectra across the extracts from *Uraria crinita* that were processed at different oven temperatures.

Metabolites	Normalized integral value (NMR signal × 10^3^)^a^		
	Oven 40	Oven 55	Oven 70
**Amino acids**			
Isoleucine	148.19 ± 16.63c,d	105.97 ± 12.37^d^	100.92 ± 10.81^c^
Valine	157.85 ± 18.47	130.66 ± 16.65	126.38 ± 9.5
Threonine	428.92 ± 31.18c,d	351.81 ± 23.44^d^	338.16 ± 22.63^c^
Alanine	636.11 ± 54.77^c^	608.61 ± 41.08	561.4 ± 20.17^c^
Arginine	1109.5 ± 130.58c,d	622.57 ± 51.89^d^	616.27 ± 33.47^c^
γ-Aminobutyric acid (GABA)	273.28 ± 41.93c,d	376.12 ± 38.8^d^	364.82 ± 13.23^c^
Proline	275.52 ± 43.73c,d	388.99 ± 48.62^d^	416.62 ± 29.7^c^
Aspartate	754.08 ± 149.08c,d	316.24 ± 40.12^d^	326.04 ± 11.03^c^
Asparagine	2401.49 ± 239.57 cd	2116.24 ± 146.21^d^	2060.3 ± 146.78^c^
Tyrosine	100.84 ± 20.28c,d	33.68 ± 4.42^d^	30.96 ± 5.81^c^
Tryptophan	63.45 ± 9.51c,d	21.52 ± 5.49^d^	18.63 ± 3.89^c^
Phenylalanine	23.44 ± 5.13c,d	11.8 ± 1.5^d^	11.88 ± 1.29^c^
**Organic acids**			
Salicylic acid	80.55 ± 19.42c,d	27.54 ± 4.6^d^	27.53 ± 3.26^c^
Acetate	777.83 ± 101.34^d^	616.71 ± 53.75^d^	699.62 ± 55.02
Formate	25.45 ± 22.53	21.39 ± 2.15	24.8 ± 2.45
**Sugars**			
Glucose	256.6 ± 39.13c,d	138.71 ± 30.61^d^	111.79 ± 5.74^c^
Sucrose	77.90 ± 16.97c,d	321.47 ± 9.44^d^	356.25 ± 13.33^c^
Pinitol	3062.8 ± 270.8	2879.45 ± 130.16	2944.64 ± 106.18
Myo-inositol	2829.38 ± 99.04	3028.6 ± 134.19	3119.85 ± 107.15
**Nucleosides**			
Adenosine	9.42 ± 1.81c,d	24.31 ± 1.03b,d	26.54 ± 2.04b,c
**Amines**			
Choline	779.35 ± 119.08c,d	1239.14 ± 61.01^d^	1242.67 ± 57.51^c^
Betaine	953.93 ± 183.39c,d	173.46 ± 18.33^d^	178.22 ± 28.46^c^
**Secondary metabolites**			
Triterpenoid (with an olean-12-ene skeleton)	623.31 ± 154.46c,d	292.65 ± 52.92^d^	275.17 ± 49.51^c^
Apigenin glycosides (27 + 28)	461.52 ± 97.94 cd	189.01 ± 30.75^d^	167.04 ± 29.09^c^
Spatholosineside A	81.99 ± 9.09c,d	29.18 ± 4.53^d^	27.36 ± 3.97^c^

^a^The results are shown as means ± SE. The statistical analysis was performed using the independent sample *t*-test and one-way ANOVA, followed by Bonferroni’s *post hoc* test. The criterion used for statistical significance was *p* < 0.05.

^b^*p* < 0.05, Oven 55 versus Oven 70; ^c^*p* < 0.05, Oven 40 versus Oven 70; ^d^*p* < 0.05, Oven 40 versus Oven 55.
