# Supplementary material for: Improving the Concentrations of the Active Components in the Herbal Tea Ingredient, Uraria crinita: The Effect of Post-harvest Oven-drying Processing
Source: Sci Rep. 2017 Jan 12;7:38763. doi: 10.1038/srep38763 (PMC5227699; doi:10.1038/srep38763)
Supplement: Supplementary Data [file srep38763-s1.doc]

Improving the Concentrations of the Active Components in the Herbal Tea Ingredient, *Uraria crinita*: The Effect of Post-harvest Oven-drying Processing

*Jung Chao* ^a†^*, Yuntao Dai* ^b†^, *Hao-Yuan Cheng* ^c^, *Wing Lam*^d^, *Yung-Chi Cheng*^d^, *Ke Li* ^e^, *Wen-Huang Peng* ^f^*, Li-Heng Pao,^gh^ Ming-Tsuen Hsieh*^f^, *Xue-Mei Qin*^e*^, *Meng-Shiou Lee*^f*^

a Institute of Pharmacology, National Yang-Ming University, College of Medicine, Taipei, Taiwan

b Institute of Chinese Materia Medica, China Academy of Chinese Medical Sciences, Beijing, China

c Department of Nursing, Chung-Jen Junior College of Nursing, Health Sciences and Management, Chia-Yi, Taiwan

d Department of Pharmacology, Yale University School of Medicine, New Haven, Connecticut, United State

e Modern Research Center for Traditional Chinese Medicine of Shanxi University, Shanxi, China

f Department of Chinese Pharmaceutical Sciences and Chinese Medicine Resources, China Medical University, Taiwan

g Research Center for Industry of Human Ecology, Chang Gung University of Science and Technology, Kweishan, Taoyuan, Taiwan

h School of Pharmacy, National Defense Medical Center, Taipei, Taiwan

*Corresponding author: Dr. Xue-Mei Qin (qinxm@sxu.edu.cn) and Dr. Meng-Shiou Lee ([leemengshiou@mail.cmu.edu.tw](mailto:leemengshiou@mail.cmu.edu.tw)) †Equal contribution.

**1. Molecular Identification of the *Uraria* species**

***1.1 Collection of Uraria plant.*** Three species of *Uraria* plants, *Uraria crinita* (Voucher No. CMU-UC001, GenBank Accession No.189714.1), *Uraria picta* (UP-3) and *Uraria lagopododes* (UL-1) were collected from Nantou and Kaohsiung Counties, Taiwan to extract their DNAs and align their ITS rRNA sequences.

***1.2 DNA extraction.*** Dried leaves from the various *Uraria* samples were ground into a powder in liquid nitrogen extract the DNAs. The total DNA was purified from each sample of homogenized leaves using a genomic extraction kit (Geneaid, Taipei, Taiwan), according to the manufacturer’s instructions. The concentration of the isolated DNA was determined with a spectrophotometer (NanoVue^TM^, GE Healthcare, USA), and each sample was stored at -20°C until further use.

***1.3 DNA amplification and sequence alignment.*** The complete ITS regions of the three *Uraria* species were amplified by PCR using the TCM-5(5’-cgtaacaaggtttccgtaggtgaac-3’) and TCM-12(5’-gacgcttctccagactacaa-3’) primers and the genomic DNA from the *Uraria* plants was used as the DNA1 template. To sequence the ITS DNA, each PCR product was individually purified for DNA sequencing. The ITS sequence of each *Uraria* species was then obtained, *Uraria crinita* (Voucher No. CMU-UC001, GenBank Accession No.189714.1), *Uraria picta* (UP-3) and *Uraria lagopododes* (UL-1) and two reference sequences, *Uraria picta* (GenBank Accession No.JF769488) and *Uraria lagopodoides* (GenBank Accession No.JF970604) were used and subjected to multiple pairwise sequence alignments using Clustal W2 software (<http://www.ebi.ac.uk/clustalw/index.html)>)***.***

***1.4 Loop-mediated isothermal amplification (LAMP) primer designation and reaction.*** Four specific LAMP primers (F3, B3, FIP and BIP) were designed using primer explorer ver. 3 software ([^http://primerexplorer.jp^](http://primerexplorer.jp/); Eiken Chemical Co. Ltd., Japan) to identify *Uraria crinita* (GenBank Accession No.189714.1) based on the sequence of the nuclear ITS2 DNA and the partial 26S ribosomal DNA. The sequence and the target position of the primers used to identify *Uraria crinita* are shown in **Figure S4A**. The LAMP reaction was performed according to the instructions in a previous report’s instructions[^47^](#_ENREF_47). In brief, a 25 microliter reaction mixture containing LAMP reaction buffer, 8 U of *Bst* DNA polymerase (NEW ENGLAND BIOLABS, GERMANY), 10 μmole/L of each of the B3 and F3 primers, 10 μmole/L of each of the BIP and FIP primers was used. Finally, 1 ng of the total *Uraria crinita* DNA was added to the LAMP reaction. The reactions were incubated in a 65°C heating block for one hour.

***1.5 Results and Discussion.*** To allow the molecular identification of *Uraria crinita* for quality control, a rapid molecular detection of *Uraria crinita* was established here using the novel nucleic acid amplification-loop-mediated isothermal amplification (LAMP) method. First, the sequence of nuclear ITS2 and 26S ribosomal DNAs were aligned and compared between the various *Uraria* species including *Uraria crinita*, *Uraria picta* and *Uraria lagopododes*, as illustrated in the upper panel of **Figure S4A**. The alignment shows that the nuclear ITS2 and 26S ribosomal DNAs of *Uraria crinita* are highly similar to those of *Uraria picta* and *Uraria lagopododes*. However, at least eight nucleotides within the *Uraria crinita* DNA sequence in these regions were different from *Uraria picta* and *Uraria lagopododes*. Therefore, based on these nucleotides the differences in the ITS2 and 26S ribosomal DNAs of the *Uraria* species, specific LAMP primers for *Uraria crinita* were designed to allow its rapid molecular identification (**Figure S4A**, lower panel). As illustrated in lane 1 of **Figure S4B**, when the LAMP reaction was performed using the *Uraria crinita*-specific LAMP primers, the resulting LAMP product, namely a ladder-like series of DNA fragments on agarose gel electrophoresis, was only present when the *Uraria crinita* DNA was used. The genomic DNAs from *Uraria picta* and *Uraria lagopododes* were not amplified by the *Uraria crinita*-specific LAMP reaction. Moreover, the ladder-like DNA of the LAMP product was also confirmed by sequencing and corresponded with the sequence of the original target, *Uraria crinita*. In conclusion, not only have we established the ITS2 sequence alignment of the *Uraria* species, but we also established a LAMP assay for the rapid detection of *Uraria crinita*. This is useful basic information, and the approach will be a very valuable and rapid tool for the molecular identification of *Uraria crinita* during quality control.

**2. GC-MS analysis**

***2.1 Sample preparation for GC-MS.*** The samples were prepared for the GC-MS metabolomics analysis using a previously described method3, with slight modifications. Fifty mg of powdered UC material were ultrasonically extracted for 30 min at 300K with 4 mL of a methanol-water-chloroform solvent (1:1:2, v/v). After a 30 min ultrasonic extraction, the tube was centrifuged at 3,000 rpm for 30 min, and each solution was separated into an upper methanol/water phase and a lower chloroform phase. The 1.5 mL upper phase was transferred into a fresh 1.5-mL tube. The methanol/water phase was evaporated and dried under a stream of nitrogen at room temperature without heating.

***2.2 Derivatization.*** During derivatization, 40 μl of the methoxyamination reagents (methoxyamine hydrochloride at 20 mg/mL in pure pyridine, prepared freshly) were added to the dried extract and vortexed; the mixture was then incubated at 80 °C for 1 h. The samples were cooled to room temperature and 50 μL of MSTFA was added to the mixtures. The mixtures were incubated at 100 °C for 40 min. Finally, 700 μL of heptane containing 0.1 mg mL/1 of tetracosane, which was used as an internal standard, were added to each sample and mixed by vortexing for 1 min before the GC analysis.

***2.3 GC-MS instrument parameters.*** The GC-MS analysis of the samples was performed on a Polaris Q ion trap mass spectrometer (Thermo Fisher Scientific Inc., USA). A DB-5MS capillary column (30 m × 250 μm i.d., 0.25 μm film thickness; 5% diphenyl cross-linked 95% dimethylpolysiloxane; Agilent J&W Scientific, Folsom, CA) was used for the GC-MS analysis. The helium carrier gas was used at a constant flow rate of 1 mL/min. Approximately 0.2 μL of the derivatized samples werre injected into the GC-MS instrument at 280 °C in split mode with the split ratio adjusted to 1:20. The interface and ion source temperatures were set at 280 °C and 200 °C, respectively. The column temperature was initially isothermal for 1 min at 50 °C, which was followed by a 3 °C/min ramp to 210 °C, and this temperature was maintained for 1 min. Then, the column temperature was increased to 280 °C at 7 °C/min for 5 min. The solvent delay was set as 4 min. The electron energy was 70 eV, and the mass data were collected in a full-scan mode (m/z 60-800).

***2.4 Identification of the GC-MS peaks*.** The peaks of the latent metabolites were identifed by comparing their mass spectra with those of the NIST 11 (National Institute of Standards and Technology, FairCom Co., USA) mass spectral library. The sugars were confirmed using standard compounds.

**Supplementary figures**

**Figure S1 The traditional drying methods used in the post-harvest processing of fresh *Uraria crinita* produce.** (A) Oven-drying; (B) Sun-drying; (C) Dried in the shade (Air-drying).


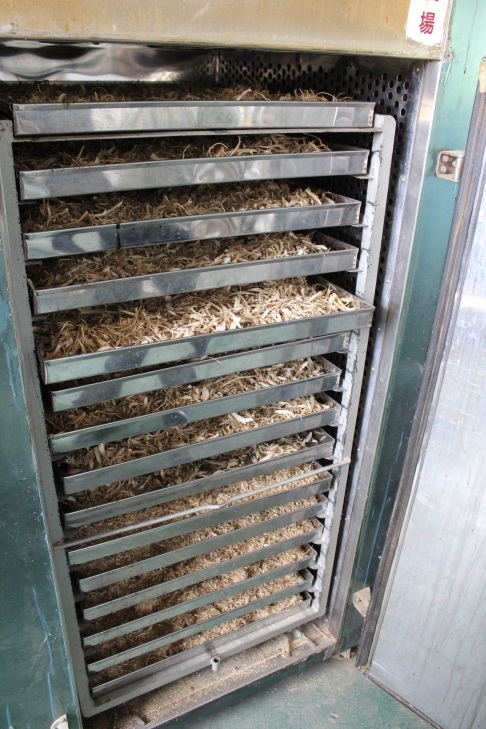

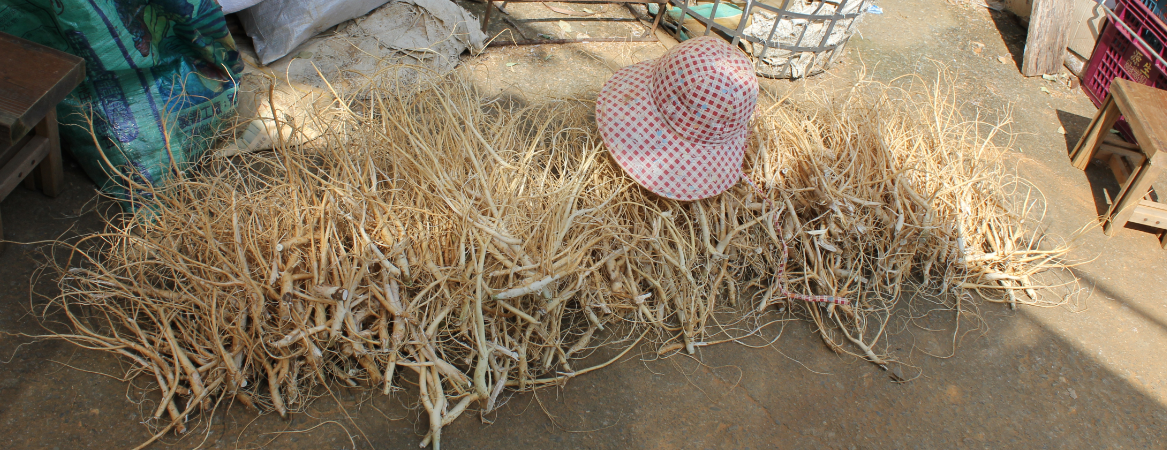
**(A) (B)**

**(C) (D)**

**
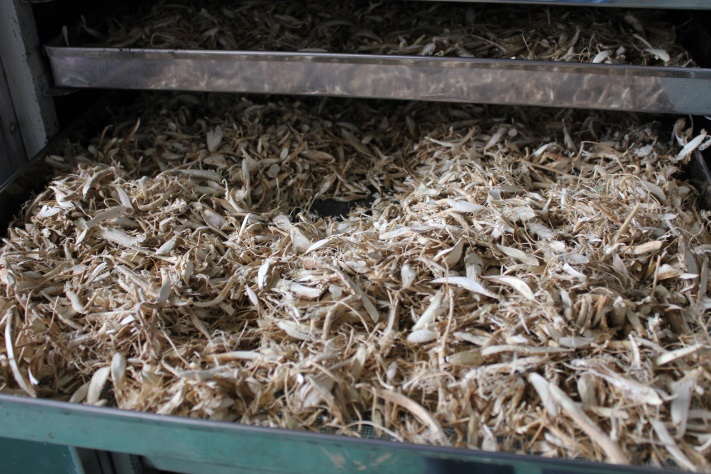

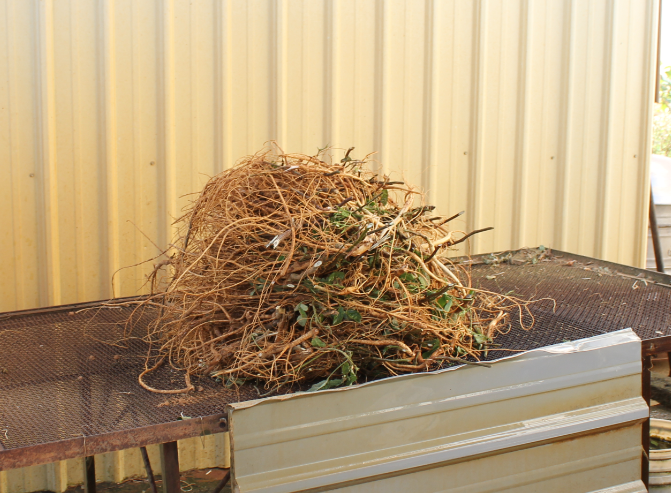
**

**Figure S2 Molecular identification of *Uraria crinita*.** (A) The ITS1 sequence of *Uraria crinita* and 4 specific primers designed for loop-mediated isothermal amplification. (B) Assay of the specificity of the loop-mediated isothermal amplification on *Uraria crinita* and its adulterants in the markets.

**(A)**

**(B)**
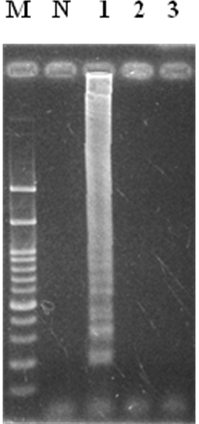


**Figure S3 The chemical structure of some secondary metabolites of *Uraria crinita*.** (1, S patholosineside A; 2 Apigen 6-C-*β*-D-apiofuranosyl (1→2)-*α*-D-xylopyranoside; 3 salicylic acid; and 4 vitexin).

**Figure S4 PLS-DA of the ^1^H NMR data for the extracts of *Uraria crinita* that were obtained from the Oven-drying group and Shade (Air-drying) group (A and B) and Oven-drying group and Sun group (C and D).** (A) and (C) are the scatter plots of the PLS-DA scores (left). (B) and (D) are the plots of the permutation test (200 permutations). The Y-axis shows the R^2^Y (green filled dots) and Q^2^Y (blue filled square) values of every model, whereas the X-axis indicates the correlation coefficient between original and permuted data response. The Y intercepts of the plot for the R^2^Y and Q^2^Y in every model are expressed as numbers. Nor, normal group; HFD, high fat diet group.


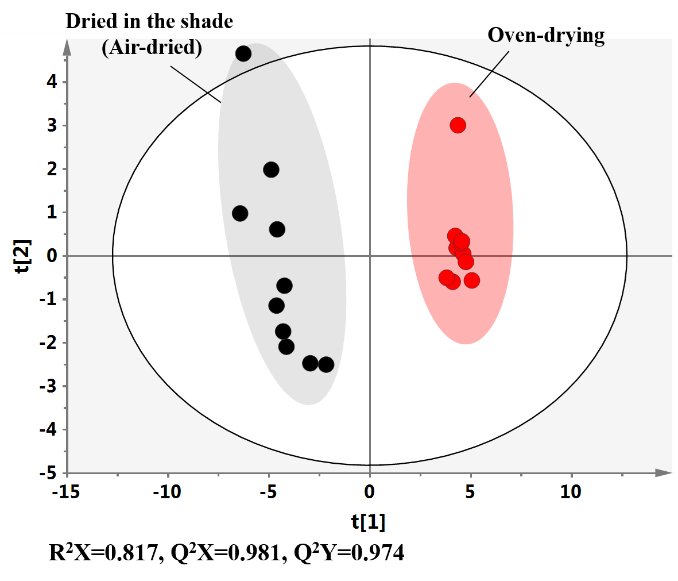
(A) (B)


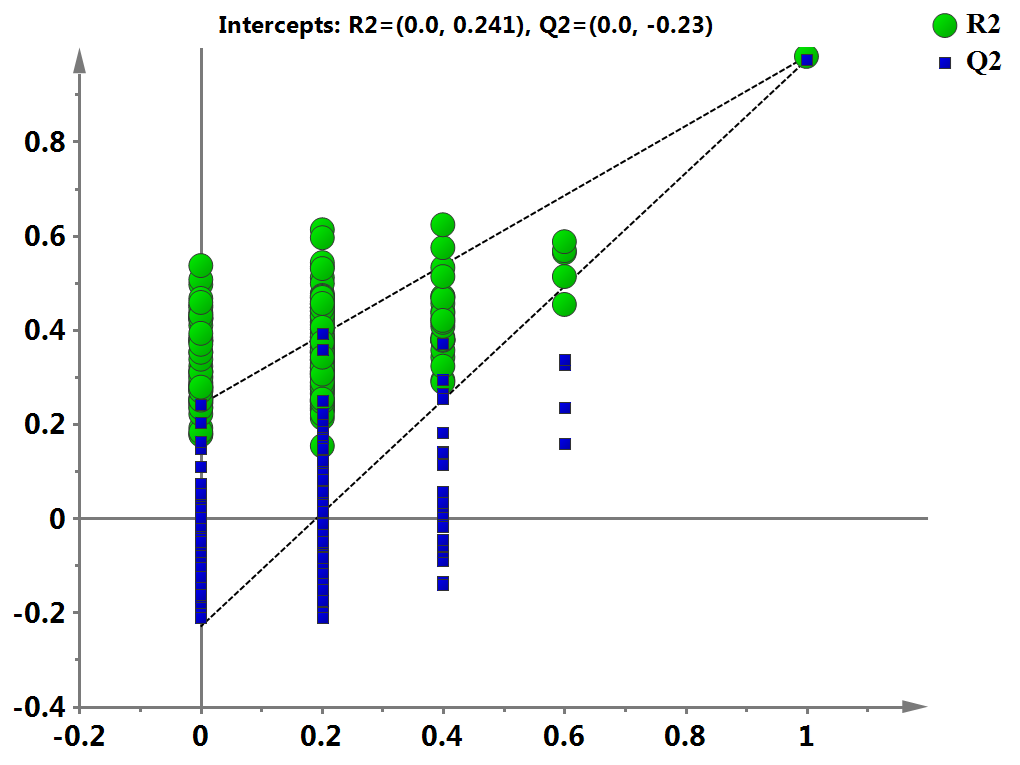


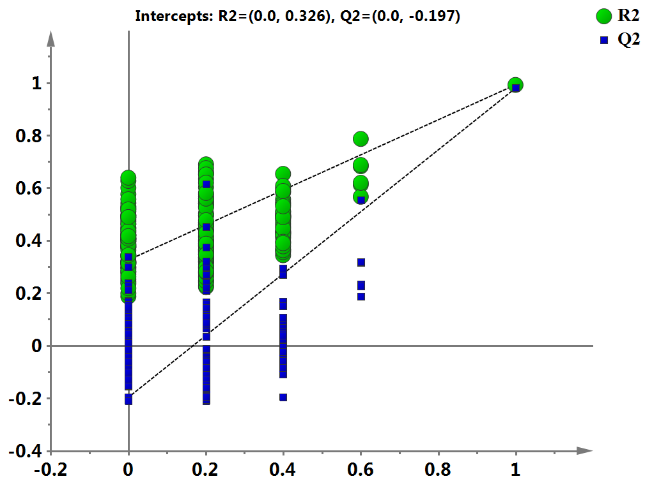

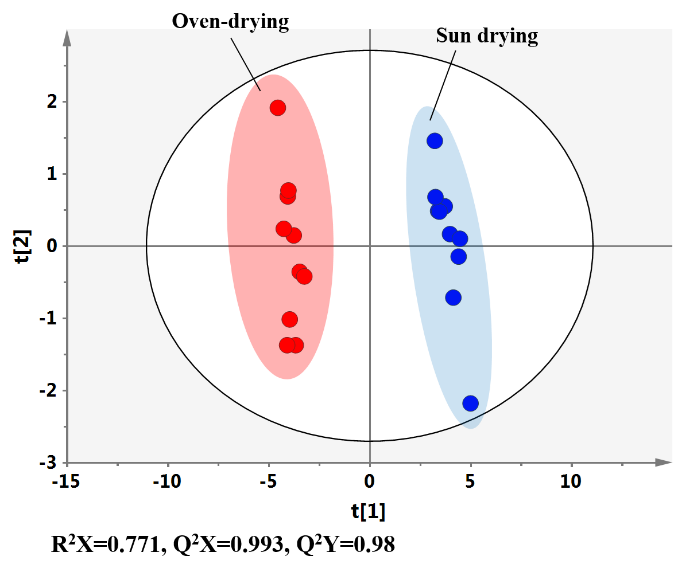
(C) (D)

**Figure S5 The 600 MHz ^1^H NMR spectra of extracts of the roots of *Uraria crinita* processed with Oven-drying at 40°C, 55°C and 70°C.**

# **11 Supplementary tables**

**Table S1 Identification of the GC-MS metabolites**

| **Compound** | **GC-MS**  **Retention time (min)** | **The M/z of the Main fragments** |
| --- | --- | --- |
| **Amino acids** |  |  |
| Valine | 9.6 | 73, 100, 144, 218 |
| Isoleucine | 10.7 | 73, 147, 158, 218, 232 |
| Leucine | 10.4 | 73, 158, 160, 232 |
| Threonine | 12.00 | 73, 147, 203, 218, 291 |
| Alanine | 7.9 | 73, 116, 147, 190 |
| γ-Aminobutyric acid (GABA) | 13.77 | 73, 86, 174, 216 |
| Proline | 10.74 | 73, 75, 142, 216 |
| Aspartate | 13.67 | 73, 100, 147, 232 |
| Asparagine | 15.49 | 73, 116, 147, 231 |
| Lysine | 12.9 | 73, 172, 244 |
| **Organic acids** |  |  |
| Acetate | 7.47 | 73, 133, 147 |
| Formate | 4.66 | 73, 131, 147, 191 |
| Citrate | 17.79 | 73, 147, 273 |
| Succinate | 13.28 | 73, 133, 147, 233 |
| Lactate | 7.25 | 73, 147, 191 |
| **Sugars and sugar alcohols** |  |  |
| Glucose | 19.4 | 73, 147, 217, 319 |
| Fructose | 19.11 | 73, 147, 217, 307 |
| Sucrose | 32.6 | 73, 147, 169, 271, 361 |
| Pinitol | 18.17 | 73, 133, 147, 217 |
| Myo-inositol | 23.38 | 73, 147, 197, 217, 265, 305, 318 |

**Table S2 Cross-validation analysis of the PLS-DA and OPLS-DA results derived from the NMR data for the extracts of *Uraria crinita* obtained from the three different drying methods.**

| **Groups** | **Permutation test *^a^*** | |  | **CV-ANOVA** |
| --- | --- | --- | --- | --- |
|  | **R^2^Y-intercept** | **Q^2^Y-intercept** |  | ***p*-value** |
| Oven versus Shade | 0.241 | -0.23 |  | 1.50322e-011 |
| Oven versus Sun | 0.326 | -0.197 |  | 2.59085e-015 |
| *^a^* The results were obtained from 200 random permutations. | | | | |

**Table S3 Cross-validation analysis of the PLS-DA and OPLS-DA results derived from the NMR data for the extracts of *Uraria crinita* obtained from the different Oven-drying temperatures.**

| **Groups** | **Permutation test *^a^*** | |  | **CV-ANOVA** |
| --- | --- | --- | --- | --- |
|  | **R^2^Y-intercept** | **Q^2^Y-intercept** |  | ***p*-value** |
| 40 °C versus non-40 °C | 0.197 | -0.198 |  | 1.44876e-017 |
| *^a^* The results were obtained from 200 random permutations. | | | | |
